# Biosensor Development and Innovation in Healthcare and Medical Applications

**DOI:** 10.3390/s23052717

**Published:** 2023-03-02

**Authors:** David K. Mills, Gergana G. Nestorova

**Affiliations:** 1 The School of Biological Sciences, Louisiana Tech University, Ruston, LA 71272, USA; 2 Molecular Science and Nanotechnology, Louisiana Tech University, Ruston, LA 71272, USA

## 1. Introduction

The pandemic necessitated a change to the historical diagnostics model. It accelerated a shift to a hybrid approach that includes an analysis of patient samples collected in distributed settings, such as at the point of care and in workplaces, schools, and community centers. Perhaps the most radical change in response to the pandemic was the large-scale production and distribution of over-the-counter SARS-CoV-2 testing technologies that could be purchased. It produced a foundational shift in the approaches of nations and their health systems for detecting and diagnosing diseases and conditions, especially infectious diseases. 

The demand for diagnostic, monitoring, and measuring sensors with clinical applications is growing [1]. The development of affordable, easy-to-use, and accurate medical devices to provide timely and actionable health status information remains critical. New healthcare delivery models foresee an empowered patient engaged in informed decision-making and care management in partnership with physicians, nurses, and healthcare practitioners. This model requires an innovation in medical device technologies that overcome the delay issues using centralized, lab-based approaches, with the patient-to-provider interaction occurring at the point of care. The near future will see the development of diagnostic systems that can help predict and stop the spread of disease. Molecular-level sensor detection networks that provide real-time pathogens or pollution monitoring of the agricultural, animal, environmental, and health fields will radically improve global healthcare.

New biosensors are needed for diagnostic tools for evaluating patient samples, such as blood, saliva, and urine in non-laboratory settings, and real-time patient data [2,3]. In addition, monitoring devices for the home-based management of chronic conditions and communication technologies enable data sharing and team-based care approaches across settings. Microphysiological analysis platforms predict the most effective treatment of diseases and provide an ideal model to address fundamental questions of development and disease pathogenesis [3,4]. They will provide a significant stimulus for new proactive, predictive, and preventive medical devices. We predict that diagnostics will become tightly integrated into treatment strategies [5,6].

These devices can expand the capabilities of primary care physicians, nurses, pharmacists, and other health care practitioners, as well as patients, to rapidly determine and deliver the appropriate treatments and prevention strategies. These smart devices will save patients’ lives, assist in controlling bacterial and viral outbreaks, and reduce healthcare costs.

Point-of-care (POC) technologies involve the use of portable devices to medically examine a patient during a consultation. POC devices can provide instantaneous results, enabling patients to receive better-informed healthcare information. POCs also provide an opportunity to deliver healthcare in low-resource settings, such as in low- and middle-income countries or disaster environments, where there is often a lack of diagnostic and monitoring tools. 

The combination of highly sensitive biosensor designs with microfluidics has increased in various lab-on-a-chip devices. These devices can reduce sample volume and detection time and improve sensitivity with high throughput operation [7].

With this in mind, the goal of this Special Issue of the Journal Sensors, belonging to the section “Biosensor Development and Innovation in Healthcare and Medical Applications”, was to collect original research manuscripts which describe cutting-edge developments in biosensor technology for medicine and clinical translational applications, as well as reviews that provide an update on the latest progress in this field.

A total of seven manuscripts have been accepted for publication. The final collection includes five original research manuscripts and two reviews by authors from several different countries. A quick overview and general classification of the manuscripts are provided below.

## 2. Contributions

(1). “DE-PNN: Differential Evolution-Based Feature Optimization with Probabilistic Neural Network for Imbalanced Arrhythmia Classification” was authored by Amanah Nasim and Yoon Sang Kim. A heartbeat classification method was developed based on evolutionary feature optimization using differential evolution (DE) and classification using a probabilistic neural network (PNN) to discriminate between normal and arrhythmic heartbeats. The proposed method follows four steps: (1) preprocessing; (2) heartbeat segmentation; (3) DE feature optimization; and (4) PNN classification and employed direct signal amplitude points constituting the heartbeat acquired from the ECG holter device with no secondary feature extraction steps. The proposed DE-PNN scheme can provide better classification accuracy considering 8 classes with only 36 features optimized from a 253-element feature set, thus implying an 85.77% reduction in direct amplitude features. Our proposed method achieved overall 99.33% accuracy, 94.56% F1, 93.84% sensitivity, and 99.21% specificity.

(2). The article, “Wearable E-Textile and CNT Sensor Wireless Measurement System for Real-Time Penile Erection Monitoring”, was authored by Yongki Heo, Jinhyung Kim, Cheolung Cha, Kyusik Shin, Jihyoung Roh, and Jungki Jo. Erectile measurements are an important indicator of male urological disease diagnosis, treatment, and results. Rigiscan has been widely used in studies and diagnoses for evaluating nocturnal erectile dysfunction during sleep. There are some limitations to this technique. In this study, we used a real-time wearable monitoring system that can quantitatively measure the length and circumference of the penis using electronic textiles (E-textile) and carbon nanotube (CNT) sensors. The E-textile sensor was used to measure the length, circumference, and gradient with portability, convenience, and comfort. The results of this study call for supplementary sensor development coupled with new technologies or existing methods for measuring erection function.

(3). “Open Software/Hardware Platform for Human-Computer Interface Based on Electrooculography (EOG) Signal Classification” was authored by Jayro Martínez-Cerveró, Majid Khalili Ardali, Andres Jaramillo-Gonzalez, Shizhe Wu et al. They discuss electrooculography (EOG) signals and Human–Computer Interfaces (HCI) for classifying four directions of eye movements employing EOG signals. The system is based on open-source ecosystems, the Raspberry Pi single-board computer, the OpenBCI biosignal acquisition device, and an open-source python library. The design is inexpensive, compact, and transportable. Their classification system can be used for input into an HCI and new assistive technology for assisted communication in paralyzed people.

(4). “Hydration Assessment Using the Bio-Impedance Analysis Method”, authored by Reem AlDisi, Qamar Bader, and Amine Bermak, discusses the bio-impedance analysis technique for the measurement of skin hydration. The study simulated a human skin model and considered the change in dielectric properties for hydration and dehydration and the frequency of the applied signal. Impedance measurements were performed using silver ink jet-printed electrodes. Simulations were performed to investigate the relationship between skin mode, the electrode design, and the measured parameters. The skin model considered the change in the dielectric properties of the skin based on the hydration status and the frequency of the applied signal. The experimental study measured and analyzed the resistance, capacitance, and phase change. The measurements for hydrated and dehydrated skin display the distinguishable difference that can be used for a non-invasive assessment of hydration levels. 

(5). “Parametric Study of Bolt Clamping Effect on Resonance Characteristics of Langevin Transducers with Lumped Circuit Models” was authored by Jinhyuk Kim and Jungwoo Lee. They developed a numerical model to analyze the resonance characteristics of Langevin transducers and experimentally compare them with corresponding experimental data, regarding the input electrical impedance and effective electromechanical coupling coefficient for the transducer at resonance modes. The experimental and theoretical values of the resonance and anti-resonance frequencies and impedance differences were closely matched. The work presented in this study provides guidelines for the pre-loading conditions of intended resonance characteristics of Langevin transducers. 

(6). “Situation Awareness-Oriented Patient Monitoring with Visual Patient Technology: A Qualitative Review of the Primary Research”, authored by David Werner Tscholl, Julian Rössler, Sadiq Said, Alexander Kaserer, Donat Rudolf Spahn, and Christoph Beat Nöthiger, discusses the current state of research on the Visual Patient technology, of which is a situation-awareness-oriented patient monitoring approach. This review provides a historical context of the patient monitoring approach and discusses its limitations. Clinical studies were discussed to assess the effects of Visual Patient technology on outcomes closely related to the concept of situation awareness. In multiple computer-based laboratory studies, Visual Patients transferred more information per unit of time with a reduced subjectively perceived workload and increased diagnostic certainty. 

(7). “Advances in Biosensors Technology for Detection and Characterization of Extracellular Vesicles”, authored by Saif Mohammad Ishraq Bari, Faria Binte Hossain, and Gergana G. Nestorova, discusses the most recent advancement in lab-on-a-chip biosensing for the isolation, detection, and characterization of extracellular vesicles. The review summarizes the principle of operation, sensitivity, and specificity of fluorescence-based, colorimetric, magnetic, surface plasmon resonance, electrochemical, and immunoaffinity sensors for the detection and molecular characterization of extracellular vesicles. 

## 3. Conclusions

In conclusion, we are pleased to serve as editors on this Special Issue of which is focused on biosensors for analytical, diagnostic, and predictive functions. We hope our efforts and the authors’ insightful manuscripts may assist the readers in conceiving new directions in biosensor development. 

Biosensors have been widely employed as they are cost-effective, provide data quickly, enable in situ placement, and provide real-time analytical data. There is an urgent need for their use in the detection of monitoring for air, water, soil pollutants, toxins, endocrine-disrupting chemicals, precision agriculture, and for monitoring climate change and its impacts.
